# Rate of Progression in Activity and Participation Outcomes in Exercisers with Parkinson's Disease: A Five-Year Prospective Longitudinal Study

**DOI:** 10.1155/2019/5679187

**Published:** 2019-09-22

**Authors:** Stephanie A. Miller, Mindy Mayol, Elizabeth S. Moore, Audra Heron, Victoria Nicholos, Brian Ragano

**Affiliations:** ^1^University of Indianapolis, Krannert School of Physical Therapy, 1400 E. Hanna Ave., Indianapolis, IN 46227, USA; ^2^University of Indianapolis, Interprofessional Health and Aging Studies, 1400 E. Hanna Ave., Indianapolis, IN 46227, USA; ^3^University of Indianapolis, Exercise Science, Department of Kinesiology, Health and Sport Sciences, 1400 E. Hanna Ave., Indianapolis, IN 46227, USA

## Abstract

**Background:**

Rates of progression of motor symptoms and physical performance show declines between 2% and 7% annually in community samples with Parkinson's disease (PD). However, the effects of ongoing exercise behaviors on progression rates have not been considered.

**Objective:**

The primary purpose of this prospective, longitudinal study was to examine the annual rates of progression in activity and participation measures over five years in community-based exercisers with PD.

**Methods:**

A cohort of 55 regular exercisers with idiopathic PD was assessed at baseline and 1, 2, and 5 years. Regular exercise was defined as scores of 4-5 on the Stages for Readiness to Exercise Scale and a self-reported average of at least 60 minutes of exercise/week within six months of each testing session. Unadjusted and adjusted annual progression rates for activity and participation measures were calculated with a standardized equation of change from baseline. A linear mixed model with covariates of age at PD diagnosis and PD subtype was used to determine adjusted change scores.

**Results:**

Annual progression rates for unadjusted and adjusted variables were similar, and none exceeded 1.7% across time points for this group of exercisers with PD. Older age at PD diagnosis significantly contributed to faster progression of walking and balance functions. A nonlinear trajectory of the PD progression was demonstrated across most activity and participation outcomes.

**Conclusions:**

Annual progression rates demonstrated by this sample of exercisers were lower than those previously reported for motor decline in general samples with PD. Assessing activity and participation outcomes longitudinally at interim time points was important for understanding the trajectory of change over time. The lower rates of progression in this study warrant further investigation into the long-term effects of exercise in PD.

## 1. Introduction

Parkinson's disease (PD) is the second most common degenerative neurologic disorder worldwide [1]. It is characterized by progressive decline in motor and nonmotor symptoms leading to increased disability and reduced quality of life. Despite a gradual loss of function with time, variations in the clinical progression of PD exist [2].

Numerous studies have explored rates of progression (i.e., worsening of symptoms) in motor symptoms and physical performance in community-based samples with PD [2–8]. In general, evidence demonstrates mean annual rates of progression between 2% and 7% as assessed with measures including the Unified Parkinson's Disease Rating Scale (UPDRS), activity of daily living (ADL) II and motor III subsections, Hoehn and Yahr scale (H&Y), and Schwab and England scale [2, 3, 5, 7]. The variability in progression rates may, in part, stem from different research methods used across studies to collect data and calculate results, with cross-sectional designs likely inflating rates of progression [9]. A number of demographic and PD-specific factors have been described as being predictive of greater disease progression across studies [2, 3, 7, 10–12]. The strongest evidence indicates that older age at disease onset and postural instability/gait difficulty (PIGD) subtype are most predictive of faster progression [11]. Despite recent emphasis on the importance of exercise and its potential to be neuroprotective for persons with PD [13], the influence of exercise behavior on PD progression has not been considered in previous studies.

The positive effects of exercise for persons with PD have been reported in a number of systematic reviews and metaanalyses [14–20]. Overall, these studies indicate that different modes of exercise, including aerobic exercise, balance activities, and resistance training, improve physical functioning and quality of life in persons with PD. The drawback is that most exercise trials included within metaanalyses are short-term exercise programs and thus do not indicate the effects of continued participation in exercise over longer periods of time. Regular exercise (>150 minutes/week) is associated with less progression of PD symptoms over one year compared to those who exercise less or not at all [21]. After two years, regular exercisers have less decline of mobility and better perception of health-related quality of life compared to nonexercisers [22]. However, this evidence is limited to retrospective data and does not extend beyond these time points. Several prospective, structured exercise trials have reported significant improvements over two years in activity-based outcomes in persons with PD, demonstrating the feasibility and promise of long-term engagement in exercise in this population [23, 24]. Only one study to date has examined ongoing participation over five years in a community-based exercise program with individuals with PD [25]. Despite a small sample, significant changes in physical function and activities were not apparent over time, indicating a positive effect of exercise on the progressive nature of PD. Subsequently, investigations on the long-term effects of regular exercise on the progression of PD are warranted. The purpose of this study was to prospectively examine annual rates of progression in activity and health-related quality of life measures over one, two, and five years in community-based exercisers with PD and to identify factors that predict greater progression in these exercisers.

## 2. Methods

### 2.1. Participants

Eighty-eight participants with idiopathic PD were enrolled in this prospective, longitudinal cohort study. The convenience sample was recruited from local PD-specific exercise programs and clinicians who treat individuals with neurologic conditions. Individuals were included in the study if they met the following criteria at baseline: (1) diagnosis of idiopathic PD, (2) living within the community, (3) stage 1–4 on the H&Y scale, (4) between the ages of 21 and 80 years, (5) able to follow three-step commands, and (6) able to travel to and from research sessions. Individuals were excluded if they had a preexisting neurological condition other than PD or previous brain surgery. All participants signed an informed consent document approved by the University of Indianapolis Institutional Review Board prior to each testing session.

For the purpose of this analysis, only participants who self-reported taking part in exercise on a regular basis over the course of the study were included in the analysis. Exercise was defined as any physical activity performed outside of normal daily activities. The Stages for Readiness to Exercise Scale was used to delineate regular participation in exercise. Regular exercisers scored a 4 or 5 on the stages for readiness to exercise scale (60 minutes of exercise per week beginning within or for longer than the last six months) [26]. Self-reported stages of change in exercise behavior have strong construct validity and test-retest reliability in both healthy and disabled populations [27, 28].

### 2.2. Procedures

Data were collected across six different testing sessions, once every six months over the first two years (baseline, 6, 12, 18, and 24 months) and again five years after baseline. Only data from baseline, 12 and 24 months, and five years were included in the current analysis. Participants were scheduled within 1–3 weeks from the original baseline testing date at the one-, two-, and five-year testing sessions and at a similar time of the day (am or pm). Participants were instructed to take their anti-PD medication one hour prior to their scheduled data collection start time in an attempt to test with the medication effect at peak dose. Testing sessions were conducted at a local fitness facility or a university laboratory. All testing was performed by entry-level Doctor of Physical Therapy student evaluators who were trained by the primary investigator (SCM) to follow standardized testing procedures. Prior to testing, interrater reliability for the Mini-Balance Evaluation Systems Test (Mini-BESTest) among all evaluators relative to this study was established (ICC_3,1_ = 0.96). Outcome measures were performed in a randomized order to reduce the effect of test order bias.

Age, age at PD diagnosis, gender, months since PD diagnosis, and PD subtype were collected and recorded at baseline. Participants were categorized as having either tremor dominant or postural instability/gait difficulty (PIGD) subtype based on item scores from the unified Parkinson's disease rating scale (UPDRS), parts II and III [29]. The H&Y scale and the average minutes of exercise per week were collected at each testing session. The H&Y scale (1–5) is a commonly accepted tool to classify disease severity in individuals with PD [30]. A higher score indicates greater disease severity. Weekly exercise logs were used to assist participants in tracking minutes of exercise per week. Data from the logs were used to confirm the regular participation in exercise, as well as the average minutes of exercise per week reported for the six months prior to the testing session.

### 2.3. Outcome Measures

The outcome measures spanned the activity and participation domains of the International Classification of Functioning, Disability and Health (ICF) by the World Health Organization [31]. The activity-based measures including the comfortable 10-meter walk test (CWT), 6-minute walk test (6MWT), activities-specific balance confidence (ABC) scale, Mini-BESTest, and UPDRS II are reliable and valid measures for persons with PD [32–36]. The walking tests were conducted on level surfaces in open hallways. For the CWT, participants were timed for the middle 10 meters of a 14-meter course at a self-selected comfortable pace [32, 37]. The mean time in seconds of three trials was converted to meters/second for analysis. Instructions for the 6MWT were given for participants to walk as far as possible in six minutes on a set, 60-meter course [38]. Distance walked was recorded in meters.

Participants were instructed on the ABC, a self-report assessment of balance confidence, to rate their confidence from 0 to 100 (0% = no confidence and 100% = full confidence) on 16 common activities [33, 36]. The mean of all items was calculated and reported as a percent, with a higher percentage indicating greater balance confidence. The 14 balance-related tasks on the Mini-BESTest are rated on a 3-point (0–2) ordinal scale with a maximum score of 28 [39]. A higher score indicates better balance. The UPDRS II, activities of daily living subsection, includes 13 items and is scored on a scale from 0–4, with a maximum score of 52 [40]. Lower scores indicate better ability to perform activities of daily living.

The 39-item Parkinson's disease questionnaire (PDQ-39) is a valid and reliable self-report questionnaire that measures health-related quality of life within the participation domain of the ICF in persons with PD [41]. Participants rated each item on a 5-point Likert scale related to how commonly they feel their disease influences that area of life (0 = never, 1 = occasionally, 2 = sometimes, 3 = often, and 4 = always). The summary index was used to represent overall feelings of function and well-being across all eight domains of the questionnaire [42]. A lower score on the PDQ-39 summary index indicates a better perception of health-related quality of life.

### 2.4. Data Analysis

Seventy-eight participants were self-reported, regular exercisers at baseline. Twenty-three participants were excluded from the current analysis because they either dropped out of the study after baseline, did not return for at least two additional testing sessions after baseline, or were no longer exercising at follow-up testing sessions. Reasons included are as follows: denied further testing, passed away, moved out of the area, unable to be contacted, no transportation to testing site, and medical complications that prohibited testing and/or continued exercise. Fifty-five participants were included in the final analysis (*n* = 55 at baseline, *n* = 50 at year 1, *n* = 55 at year 2, and *n* = 34 at year 5).

Assumptions for parametric statistics were assessed by examining normality of data. Descriptive statistics were conducted to illustrate the baseline characteristics of the total sample of regular exercisers, as well as the analyzed and excluded participants. Categorical data are presented as frequencies and percentages, while continuous data are reported as means and standard deviations or medians and interquartile ranges, depending on normality of data distribution. Data were analyzed using IBM SPSS 25.0 (IBM Corp., Armonk, NY) and the alpha level was set at *p* < 0.05.

Rate of progression was determined by comparing change scores from baseline to one year, baseline to two years, and baseline to five years with all percentages standardized to an annual rate of change [5, 9]. Change scores from baseline to each follow-up session across all variables were assessed for normality. All change scores were normally distributed, except for H&Y and ABC; thus, only median progression rates are reported for those measures. Annual progression rates were calculated using two approaches. First, progression rates (%) were calculated using an unadjusted, standardized equation that takes the difference between baseline and follow-up values, divided by the maximum or baseline score, multiplied by 100, and divided by the appropriate number of years from follow-up (1, 2, or 5). This calculation was restricted to only those participants with complete data at each follow-up period. Second, we used a linear mixed model (LMM) analysis to calculate the adjusted mean change scores for each outcome over time taking into account the covariates of age at PD diagnosis and PD subtype [11]. Linear mixed models are well suited for longitudinal data, as this analysis can account for autocorrelation with repeated measures, as well as missing data. A restricted maximum likelihood (REML) model was used with an autoregressive, heterogeneous covariance structure, and random intercepts. The adjusted change scores generated from the LMM analysis were then applied to the same standardized equation described above to calculate adjusted progression rates (%).

## 3. Results

Baseline descriptive statistics for the total sample, as well as analyzed and excluded cohorts are included in Table 1. Those who were excluded from the analysis had significantly higher scores on the H&Y scale, reduced gait endurance, and greater balance impairment compared to the analyzed group. There were significantly more individuals in the excluded group with PIGD subtype, which might explain why they had more difficulty with gait and balance. No other differences were found between groups. The mean (standard deviation) age at baseline of the analyzed group of participants was 66.8 (8.0) years with a mean age at PD diagnosis of 61.4 (9.6) years. The analyzed cohort had a median (interquartile range) H&Y stage of 1.0 (1.0). They regularly exercised a mean of 259.6 (112.3) minutes per week.

Unadjusted annual rates of progression calculated with the standardized equation (Table 2) demonstrated either no change or improvements across the majority of outcome measures, from baseline to year one (0–9.7%) and from baseline to year two (0–3%). Those outcomes that demonstrated a decline were not greater than 1.2% annual progression over the first two years. Data were used from year five; however, most measures showed annual rates of progression of 1.1% or less. The CWT was the only measure that demonstrated improved function compared to baseline at all three time points.

**Figure 1:**
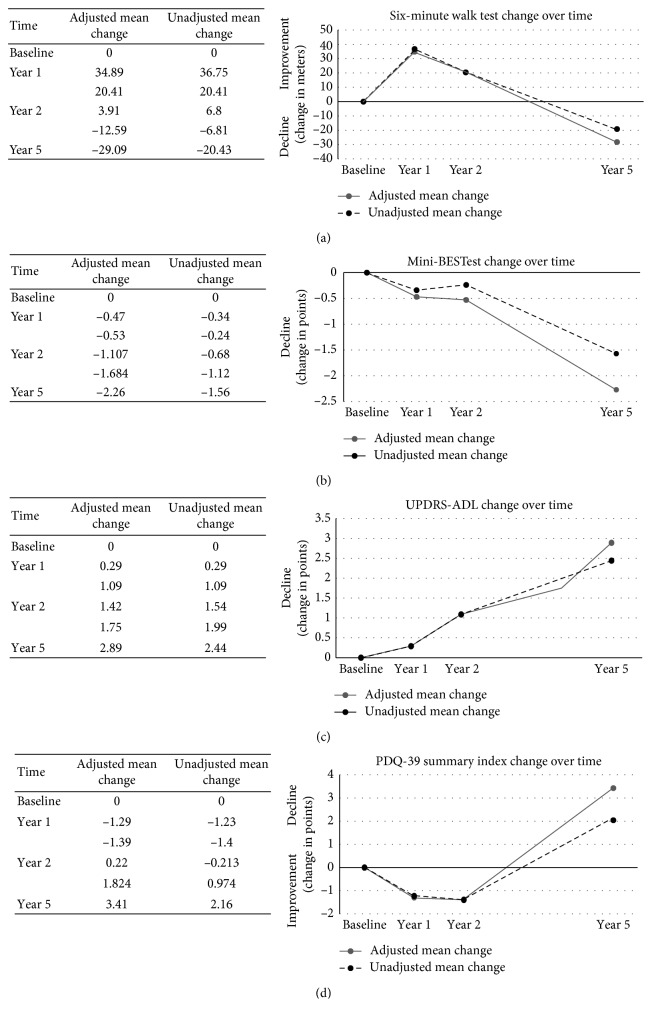
Change scores relative to baseline at years 1, 2, and 5. Grey line represents the adjusted change scores calculated from the LMM analysis (adjusted for age at PD diagnosis and PD subtype). Black dashed line represents the unadjusted, raw change scores. (a) Six-minute walk test change over time. (b) Mini-BESTest change over time. (c) UPDRS II (ADL subscale) change over time. (d) PDQ-39 summary index change over time.

With the LMM procedure, all outcomes showed significant change over time, excluding the CWT (*p*=0.07). Age at PD diagnosis significantly contributed to the CWT, 6MWT, and Mini-BESTest models, resulting in a greater decline of walking and balance function over time with older age at PD diagnosis (*p* < 0.05). The PD subtype did not significantly contribute to any models (*p* > 0.05). Adjusted progression rates calculated with change scores generated from the LMM analysis along with unadjusted progression rates are presented together graphically in Figures 1(a)–1(d). Adjusted progression rates were 1.7% or less annually over 5 years (Table 3). Distance walked on the 6MWT significantly improved by 7.3% (0.03%, 11.8% 95% CI) one year after baseline but demonstrated a significant annual decline by −1.2% (−2.4%, −0.03%, and 95% CI) over five years compared to baseline. Scores on the Mini-BESTest and UPDRS II both demonstrated significant annual declines over five years (−1.6% (−2.7%, −0.6% 95% CI) and 1.1 (0.3%, 1.9% 95% CI), respectively).

## 4. Discussion

We prospectively examined annual rates of progression of activity and participation-based outcomes across interim time points for five years in a community-dwelling group of regular exercisers with PD. The annual progression rates for all of the variables in this study, adjusted and unadjusted, did not exceed 1.7% across time points. This is less than previously reported annual progression rates for motor and physical decline that ranged from 2% to 7% in general samples with PD [2, 3, 5, 7]. Specifically, the mean annual progression rate on the UPDRS II reported by Alves et al. [3] was 3.5% over 8 years compared to only 1.1% annually over 5 years in the current study. Likewise, the H&Y scale has been found to have median progression rates of 4% over 1 year and 1.2% over 4 years compared to 0% in the current study [5]. Exercise behaviors of participants in these earlier studies were not reported or considered as a possible factor in progression rates. Given that inactivity is common in individuals with PD [43], it is assumed that many individuals in these previous studies were not regular exercisers. On the other hand, all participants in the current study regularly participated in exercise activities. Their lower rates of progression across activity and participation outcomes warrant further investigation into the long-term effects of exercise on PD motor symptoms and physical performance.

Short-term gains in motor and functional outcomes through highly structured exercise-based clinical trials for persons with PD are promising [14–20]. However, the level of supervision, mode, amount (i.e., duration and frequency), and intensity of training provided in research environments does not always reflect real-world exercise behaviors. Exercise in the current study was not standardized, encompassing any community-based physical activity outside of normal daily activities. Participants self-reported taking part in group-based exercise classes and/or individual-based exercise with modes such as walking, running, cycling, water aerobics, boxing, home videos, or general exercise. Similar to the two-year, retrospective, observational study by Rafferty et al. [22] and the five-year, prospective, exercise program by States et al. [25], our results suggest that participation in community-based exercise for an extended period of time is beneficial for people with PD. While we defined regular exercise as at least 60 minutes of exercise per week, our analyzed sample exercised a self-reported mean of 260 minutes per week at baseline, and this amount of exercise did not significantly change over the 5 years of the study (*p*=0.87). This is well above the recommended amount of weekly exercise by the American College of Sports Medicine [44] as well as above the minimum of 150 minutes per week that has been reported for less motor progression and improved quality of life [21, 22]. Future research should take into consideration how exercise parameters such as mode, amount, and intensity affect disease progression over time.

Examination of annual progression rates in most studies span the entire duration of follow-up, which may be a partial cause for the inconsistency in progression rates across the literature and lower annual progression rates in studies of longer duration [11]. Analyzing progression with both actual, raw change scores and annual percentages at different time intervals, as we conducted in the present study, may better characterize change in function over the course of the disease. Our results provide support for nonlinearity in the trajectory of PD progression [2, 4, 45]. Reinoso et al. [2], reported a nonlinear pattern of motor progression that included a period of improvement, followed by stability and eventual progression when following individuals with PD for nine years beginning early after diagnosis. Subsequently, their annual rates of progression based on UPDRS motor scores varied from 3% improvement to 2% progression depending on the phase. Most of the activity and participation measures used in the current study demonstrated similar patterns of improvement or stability over the first couple years, followed by subsequent decline at year five. It is interesting to note that participants in the current study were further from diagnosis compared to those in Reinoso et al. [2]. Yet, at a time when stability or worsening of scores would have been expected based on the group's time since diagnosis, the exercisers in our study continued to make improvements in most outcomes. All of the participants in our study were regular exercisers at baseline; however, we did not record prestudy exercise habits, such as how long they had been exercising or if they had recently started an exercise program. While evidence supports improvements in the activity and participation after initiating an exercise program in persons with PD [14–20], we can only speculate that the early improvements found in our sample were exercise-related.

We employed two longitudinal approaches to calculate change scores [9]. The unadjusted method takes into consideration two time points of data for each separate calculation and uses only complete data. This approach is simple and can be completed with a standard calculator. On the other hand, the more complex LMM analysis uses all time points within a single calculation, takes into consideration potential covariates, and accounts for missing data, inherently maintaining sample size. Despite these different statistical approaches, results from both were similar. Overall, progression rates generated from change scores with the LMM analysis were, in most instances, only slightly higher compared to progression rates computed with the unadjusted method. This has important implications for clinical practice. The standardized equation with the unadjusted method could be used by clinicians within a healthcare environment to calculate patient-specific progression rates across visits that adequately reflect their percent change over time. Monitoring rates of progression in patients with PD can be an essential tool to enhance treatment planning and long-term patient outcomes.

Age at PD diagnosis did significantly impact models for the CWT, 6MWT, and Mini-BESTest as evidenced by the LMM analysis. This is consistent with the previous findings that suggest that older age at diagnosis is associated with faster progression of levodopa-resistant motor impairments [2, 3, 7, 11]. From a clinical standpoint, the age of an individual at PD diagnosis is an important consideration when setting goals and determining anticipated outcomes, regardless of the level of physical activity or exercise. In contrast to previous reports, the PD subtype did not significantly contribute to any models indicating that PIGD dominance did not impact faster progression rates over time in this group of regular exercisers [2, 11]. Considering that postural stability and balance are improved with exercise interventions [14, 15, 20], we speculate that long-term participation in exercise by individuals in this study may have lessened the impact of the PD subtype on the outcomes.

Several limitations should be acknowledged. Thirty-eight percent of the analyzed sample did not return for follow-up at year five, potentially biasing the results and underestimating progression. In addition, due to the small sample size, we were not able to account for all potential confounders in the LMM analysis. Demographic variables, comorbidities, levodopa equivalent daily dose and nonmotor symptoms (i.e., cognition) should be considered with future, larger samples. Change in type and dosage of anti-PD medications over time was not collected, and all measures were conducted during the “ON” phase of anti-PD medications, possibly altering outcomes from natural disease progression. The measures used in the current study reflect only changes in activity and participation-based outcomes. Change in PD-specific motor symptoms, as commonly reported with the UPDRS motor scores were not collected across time in this study, limiting our ability to compare outcomes directly to other studies. Finally, due to the lack of a comparison to those who do not exercise or exercise irregularly, we cannot conclude with certainty that exercise slows the PD progression.

## 5. Conclusions

In conclusion, annual rates of progression in activity and participation-based outcomes in this sample of exercisers with PD did not exceed 1.7% at any time point. Observed changes at interim time points over an extended period of time suggest a nonlinear trajectory of PD progression with a regular participation in exercise. In addition, the results supported findings from previous studies that older age at PD diagnosis is related to a faster progression of walking and balance functions. Assessing the rate of PD progression with these commonly used activity and participation-based outcomes provides groundwork for future and long-term exercise studies.

## Figures and Tables

**Table 1:** Demographic and outcome measures at baseline for the total sample and differences between analyzed and excluded samples across baseline variables.

	Total sample mean (SD) (*n* = 78)	Analyzed sample mean (SD) (*n* = 55)	Excluded sample mean (SD) (*n* = 23)	*p*
Age (years)	66.73 (8.47)	66.84 (8.04)	66.48 (9.61)	0.87
Age at PD diagnosis (years)	61.59 (9.89)	61.36 (9.64)	62.13 (10.67)	0.76
Male sex, *n* (%)	55 (70.5)	39 (70.9)	16 (69.6)	1.00
Hoehn and Yahr stage^*∗*^	2.00 (1.00)	1.00 (1.00)	2.00 (2.00)	**0.02**
Months after diagnosis^*∗*^	62.50 (63.00)	67.00 (85.00)	54.00 (56.00)	0.66
PIGD subtype *n* (%)	29 (37)	16 (29)	13 (57)	**0.04**
Minutes of exercise per week	265.36 (142.77)	259.56 (112.27)	284.12 (217.78)	0.66
CWT (m/s)	1.23 (0.25)	1.25 (0.24)	1.17 (0.25)	0.19
6MWT (meters)	457.33 (110.90)	475.15 (110.25)	414.71 (102.55)	**0.03**
ABC (%)^*∗*^	88.75 (21.09)	88.75 (20.00)	88.75 (28.13)	0.57
Mini-BESTest^*∗*^	24.00 (6.00)	24.00 (4.00)	22.00 (6.00)	**<0.00**
UPDRS II	12.18 (5.15)	11.96 (5.29)	12.70 (4.88)	0.57
PDQ-39 summary index (%)^*∗*^	14.95 (15.53)	14.58 (13.49)	16.98 (18.07)	0.43

^*∗*^Median (IQR). PD = Parkinson's disease; PIGD = postural instability gait/difficulty; CWT = comfortable 10-meter walk test; 6MWT = six-minute walk test; ABC = activities-specific balance confidence scale; Mini-BESTest = Mini-Balance Evaluations System Test; UPDRS = unified Parkinson's disease rating scale; PDQ-39 = 39-item Parkinson's disease questionnaire.

**Table 2:** Standardized, unadjusted progression rates (in % of maximum score per year or % of baseline score per year).

	Progression rate per year		
	Over 1 year *n* = 50 Mean % (SD)	Over 2 years *n* = 55 Mean % (SD)	Over 5 years *n* = 34 Mean % (SD)
CWT^*∗*^	6.6 (19.3)	2.9 (9.5)	0.4 (5.1)
6MWT^*∗*^	9.7 (21.8)	3.0 (12.4)	−0.5 (4.1)
Mini-BESTest	−1.2 (8.5)	−0.4 (4.8)	−1.1 (2.1)
UPDRS II	0.6 (7.3)	1.1 (4.2)	0.9 (2.3)
PDQ-39 summary index	−1.2 (6.8)	−0.7 (4.0)	0.4 (2.6)
	Median % (IQR)	Median % (IQR)	Median % (IQR)
H&Y score	0.0 (20.0)	0.0 (10.0)	0.0 (4.0)
ABC	0.0 (10.7)	−0.9 (7.1)	−1.1 (2.7)

^*∗*^% of baseline score per year. CWT = comfortable 10-meter walk test; 6MWT = six-minute walk test; Mini-BESTest = Mini-Balance Evaluations System Test; UPDRS = unified Parkinson's disease rating scale; PDQ-39 = 39-item Parkinson's disease questionnaire; H&Y = Hoehn and Yahr; ABC = activities-specific balance confidence scale. A negative percentage indicates a progression (decline) in scores for the following measures: CWT, 6MWT, Mini-BESTest, and ABC. A positive percentage indicates a progression (decline) in scores for the following measures: UPDRS II, PDQ-39, and H&Y (with the opposite sign indicating improvement of symptoms).

**Table 3:** Adjusted progression rates (in % of maximum score per year or % of baseline score per year).

	Progression rate per year		
	Over 1 year % (95% CI)	Over 2 years % (95% CI)	Over 5 years % (95% CI)
6MWT^*∗*^	**7.3 (2.8, 11.8)** ^†^	2.1 (−0.5, 4.7)	−**1.2 (**−**2.4,** −**0.03)**^‡^
Mini-BESTest	−1.7 (−4.0, 0.6)	−0.9 (−2.5, 0.7)	−**1.6 (**−**2.7,** −**0.6)**^†^
UPDRS II	0.6 (−1.3, 2.5)	1.0 (−0.0, 2.1)	**1.1 (0.3, 1.9)** ^†^
PDQ-39 summary index	−1.3 (−3.3, 0.8)	−0.7 (−1.8, 0.4)	0.7 (−0.1, 1.4)

^*∗*^% of baseline score per year; ^†^*p* < 0.01; ^‡^*p* < 0.05. 6MWT = six-minute walk test; Mini-BESTest = Mini-Balance Evaluations System Test; UPDRS = unified Parkinson's disease rating scale; PDQ-39 = 39-item Parkinson's disease questionnaire.A negative percentage indicates a progression (decline) in scores for the following measures: 6MWT and Mini-BESTest. A positive percentage indicates a progression (decline) in scores for the following measures: UPDRS II and PDQ-39 (with the opposite sign indicating improvement of symptoms).

## Data Availability

The data used to support the findings of this study are available from the corresponding author upon request.

## References

[1] Pringsheim T., Jette N., Frolkis A., Steeves T. D. L. (2014). The prevalence of Parkinson’s disease: a systematic review and meta-analysis. *Movement Disorders*.

[2] Reinoso G., Allen J. C., Au W.-L., Seah S.-H., Tay K.-Y., Tan L. C. S. (2015). Clinical evolution of Parkinson’s disease and prognostic factors affecting motor progression: 9-year follow-up study. *European Journal of Neurology*.

[3] Alves G., Wentzel-Larsen T., Aarsland D., Larsen J. P. (2005). Progression of motor impairment and disability in Parkinson disease. *Neurology*.

[4] Ellis T. D., Cavanaugh J. T., Earhart G. M. (2016). Identifying clinical measures that most accurately reflect the progression of disability in Parkinson disease. *Parkinsonism & Related Disorders*.

[5] Schrag A., Dodel R., Spottke A., Bornschein B., Siebert U., Quinn N. P. (2007). Rate of clinical progression in Parkinson’s disease. A prospective study. *Movement Disorders*.

[6] Simuni T., Siderowf A., Lasch S. (2018). Longitudinal change of clinical and biological measures in early Parkinson’s disease: Parkinson’s progression markers initiative cohort. *Movement Disorders*.

[7] Velseboer D. C., Broeders M., Post B. (2013). Prognostic factors of motor impairment, disability, and quality of life in newly diagnosed PD. *Neurology*.

[8] Holden S. K., Finseth T., Sillau S. H., Berman B. D. (2018). Progression of MDS-UPDRS scores over five years in de novo Parkinson disease from the Parkinson’s progression markers initiative cohort. *Movement Disorders Clinical Practice*.

[9] Marinus J., van der Heeden J. F., van Hilten J. J. (2014). Calculating clinical progression rates in Parkinson’s disease: methods matter. *Parkinsonism & Related Disorders*.

[10] Mendes A., Gonçalves A., Vila-Chã N. (2016). Statistical models of Parkinson’s disease progression: predictive validity in a 3-year follow-up. *Journal of Parkinson’s Disease*.

[11] Post B., Merkus M. P., de Haan R. J., Speelman J. D., CARPA Study Group (2007). Prognostic factors for the progression of Parkinson’s disease: a systematic review. *Movement Disorders*.

[12] Zhao Y. J., Wee H. L., Chan Y.-H. (2010). Progression of Parkinson’s disease as evaluated by Hoehn and Yahr stage transition times. *Movement Disorders*.

[13] Zigmond M. J., Smeyne R. J. (2014). Exercise: is it a neuroprotective and if so, how does it work?. *Parkinsonism & Related Disorders*.

[14] Allen N. E., Sherrington C., Paul S. S., Canning C. G. (2011). Balance and falls in Parkinson’s disease: a meta-analysis of the effect of exercise and motor training. *Movement Disorders*.

[15] Dibble L. E., Addison O., Papa E. (2009). The effects of exercise on balance in persons with Parkinsonʼs disease: a systematic review across the disability spectrum. *Journal of Neurologic Physical Therapy*.

[16] Goodwin V. A., Richards S. H., Taylor R. S., Taylor A. H., Campbell J. L. (2008). The effectiveness of exercise interventions for people with Parkinson’s disease: a systematic review and meta-analysis. *Movement Disorders*.

[17] Shu H.-F., Yang T., Yu S.-X (2014). Aerobic exercise for Parkinson’s disease: a systematic review and meta-analysis of randomized controlled trials. *PloS One*.

[18] Tomlinson C. L., Patel S., Meek C. (2001). Physiotherapy versus placebo or no intervention in Parkinson’s disease. *Cochrane Database of Systematic Reviews*.

[19] Uhrbrand A., Stenager E., Pedersen M. S., Dalgas U. (2015). Parkinson’s disease and intensive exercise therapy—a systematic review and meta-analysis of randomized controlled trials. *Journal of the Neurological Sciences*.

[20] Klamroth S., Steib S., Devan S., Pfeifer K. (2016). Effects of exercise therapy on postural instability in Parkinson disease. *Journal of Neurologic Physical Therapy*.

[21] Oguh O., Eisenstein A., Kwasny M., Simuni T. (2014). Back to the basics: regular exercise matters in Parkinson’s disease: results from the National Parkinson Foundation QII registry study. *Parkinsonism & Related Disorders*.

[22] Rafferty M. R., Schmidt P. N., Luo S. T. (2017). Regular exercise, quality of life, and mobility in Parkinson’s disease: a longitudinal analysis of National Parkinson Foundation quality improvement initiative data. *Journal of Parkinson’s Disease*.

[23] Duncan R. P., Earhart G. M. (2014). Are the effects of community-based dance on Parkinson disease severity, balance, and functional mobility reduced with time? A 2-year prospective pilot study. *The Journal of Alternative and Complementary Medicine*.

[24] Prodoehl J., Rafferty M. R., David F. J. (2015). Two-year exercise program improves physical function in Parkinson’s disease. *Neurorehabilitation and Neural Repair*.

[25] States R. A., Sweeny T. L., Rossi A., Spierer D. K., Salem Y. (2017). Physical functioning after 1, 3, and 5 years of exercise among people with Parkinson’s disease: a longitudinal observational study. *Journal of Geriatric Physical Therapy*.

[26] Courneya K. S. (1995). Understanding readiness for regular physical activity in older individuals: an application of the theory of planned behavior. *Health Psychology*.

[27] Cardinal B. J. (1997). Construct validity of stages of change for exercise behavior. *American Journal of Health Promotion*.

[28] Cardinal B. J., Kosma M., McCubbin J. A. (2004). Factors influencing the exercise behavior of adults with physical disabilities. *Medicine & Science in Sports & Exercise*.

[29] Jankovic J., McDermott M., Carter J. (1990). Variable expression of Parkinson’s disease: a base-line analysis of the DAT ATOP cohort. *Neurology*.

[30] Goetz C. G., Poewe W., Rascol O. (2004). Movement disorder society task force report on the Hoehn and Yahr staging scale: status and recommendations The movement disorder society task force on rating scales for Parkinson’s disease. *Movement Disorders*.

[31] World Health Organization (2019). Towards a common language for functioning, disability and health: ICF International classification of functioning, disability and health. http://www.who.int/classifications/icf/en/.

[32] Combs S. A., Diehl M. D., Filip J., Long E. (2014). Short-distance walking speed tests in people with Parkinson disease: reliability, responsiveness, and validity. *Gait & Posture*.

[33] Dal Bello-Haas V., Klassen L., Sheppard M. S., Metcalfe A. (2011). Psychometric properties of activity, self-efficacy, and quality-of-life measures in individuals with Parkinson disease. *Physiotherapy Canada*.

[34] Godi M., Franchignoni F., Caligari M., Giordano A., Turcato A. M., Nardone A. (2013). Comparison of reliability, validity, and responsiveness of the mini-BESTest and Berg balance scale in patients with balance disorders. *Physical Therapy*.

[35] Leddy A. L., Crowner B. E., Earhart G. M. (2011). Utility of the mini-BESTest, BESTest, and BESTest sections for balance assessments in individuals with Parkinson disease. *Journal of Neurologic Physical Therapy*.

[36] Steffen T., Seney M. (2008). Test-retest reliability and minimal detectable change on balance and ambulation tests, the 36-item short-form health survey, and the unified Parkinson disease rating scale in people with parkinsonism. *Physical Therapy*.

[37] Flansbjer U. B., Holmbäck A. M., Downham D., Patten C., Lexell J. (2005). Reliability of gait performance tests in men and women with hemiparesis after stroke. *Journal of Rehabilitation Medicine*.

[38] American Thoracic Society (2002). ATS statement: guidelines for the six-minute walk test. *American Journal of Respiratory and Critical Care Medicine*.

[39] Franchignoni F., Horak F., Godi M., Nardone A., Giordano A. (2010). Using psychometric techniques to improve the balance evaluation systems test: the mini-BESTest. *Journal of Rehabilitation Medicine*.

[40] Movement Disorder Society Task Force on Rating Scales for Parkinson’s Disease (2003). The unified Parkinson’s disease rating scale (UPDRS): status and recommendations. *Movement Disorders*.

[41] Hagell P., Nygren C. (2007). The 39 item Parkinson’s disease questionnaire (PDQ-39) revisited: implications for evidence based medicine. *Journal of Neurology, Neurosurgery & Psychiatry*.

[42] Jenkinson C., Fitzpatrick R., Peto V., Greenhall R., Hyman N. (1997). The Parkinson’s Disease Questionnaire (PDQ-39): development and validation of a Parkinson’s disease summary index score. *Age and Ageing*.

[43] Nimwegen M., Speelman A. D., Hofman-van Rossum E. J. M. (2011). Physical inactivity in Parkinson’s disease. *Journal of Neurology*.

[44] Garber C. E., Blissmer B., Deschenes M. R. (2011). Quantity and quality of exercise for developing and maintaining cardiorespiratory, musculoskeletal, and neuromotor fitness in apparently healthy adults. *Medicine & Science in Sports & Exercise*.

[45] López I. C., Ruiz P. J. G., Del Pozo S. V. F., Bernardos V. S. (2010). Motor complications in Parkinson’s disease: ten year follow-up study. *Movement Disorders*.

